# 2730. Clinically significant cytomegalovirus infection in patients with lymphoma and no prior cellular therapy

**DOI:** 10.1093/ofid/ofad500.2341

**Published:** 2023-11-27

**Authors:** Anna Zubovskaia, Fareed Khawaja, Roy F Chemaly, Brian Primeaux, Mohammad Waleed

**Affiliations:** Baylor College of Medicine, MD Anderson Cancer Center, Houston, Texas; The University of Texas MD Anderson Cancer Center, Houston, Texas; MD Anderson, Houston, Texas; MD Anderson Cancer Center, Houston, Texas; MD Anderson Cancer Center, Houston, Texas

## Abstract

**Background:**

Reactivation of cytomegalovirus (CMV) is commonly reported in lymphoma patients who undergo cellular therapy, with often poor outcomes. The risk for CMV reactivation in patients with lymphoma prior to cellular therapy is not well understood. As new alternative immunosuppressive therapies emerge for lymphoma, understanding baseline risk for CMV reactivation is paramount to improve clinical and oncologic outcomes. Our goal was to identify risk factors, and describe outcomes associated with clinically significant CMV infection (CS-CMVi) in lymphoma patients with no prior cellular therapy.

**Methods:**

We performed a retrospective cohort study for all lymphoma patients who underwent testing for CMV by PCR from blood between 1/2017 and 12/2020. Patients with prior history of transplantation or chimeric antigen receptor therapy were excluded. Patients with and without CS-CMVi were identified for risk factor analysis by univariate analysis.

**Results:**

A total of 217 lymphoma patients were included in our analysis; CS-CMVi was identified in 41 (19%) patients. When compared to patients without CS-CMVi (control group), lymphoma patients with CS-CMVi were more likely to have history of HIV (5% vs 0%; p= 0.0350), primary central nervous system lymphoma (7% vs 1%; p= 0.0220) and relapsed disease (54% vs 25%; p= 0.0005) (Table 1). CS-CMVi was also more common if testing was done at time of relapse diagnosis (32% vs 10%; p= 0.0007), during work up for infection (98% vs 68%; p < 0.0001) and more often (3 vs 1; p < 0.0001) (table 1). Mortality and remission rates within 1 year of testing were worse in patients with CS-CMVi (table 2 and figure 1).

**Figure:**
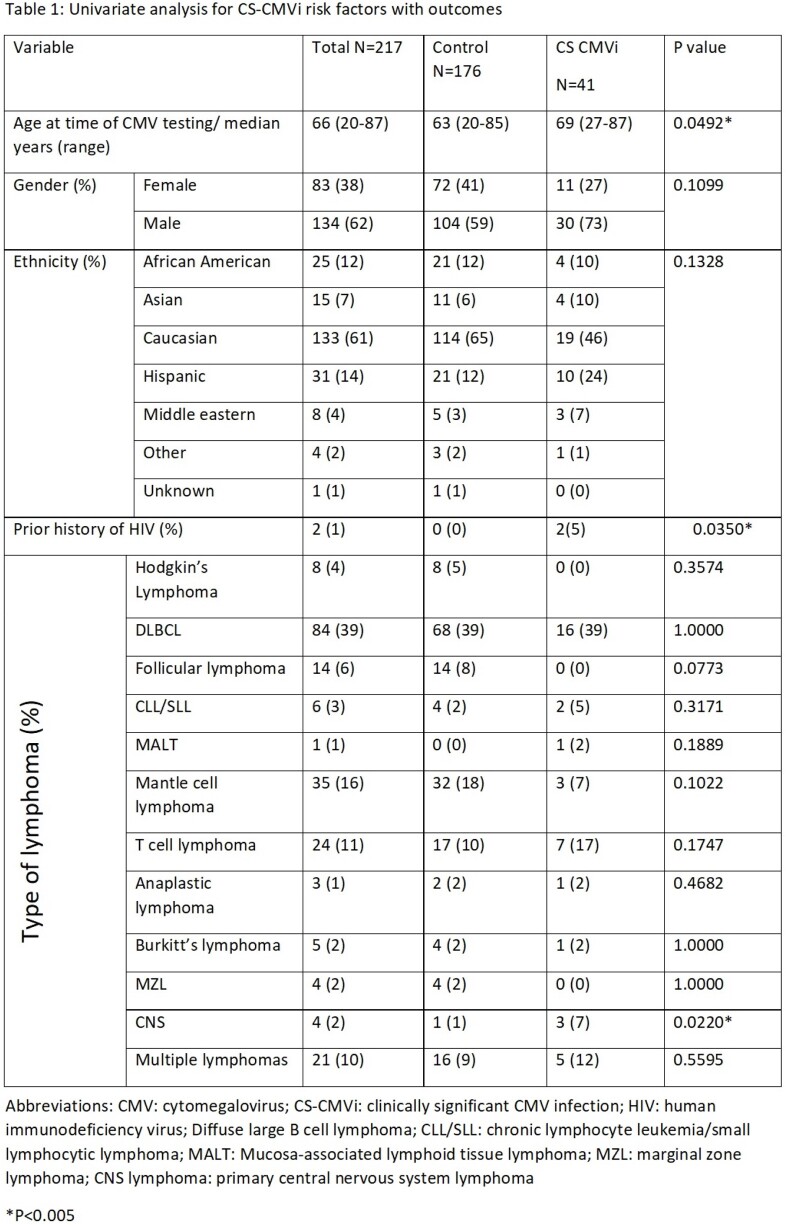

**Figure:**
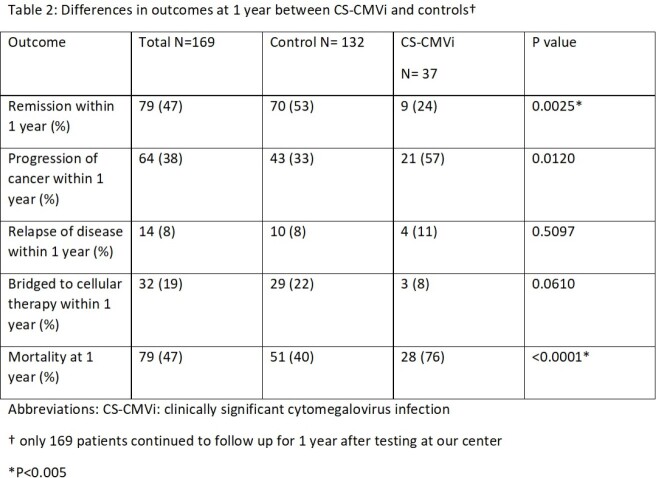

**Figure:**
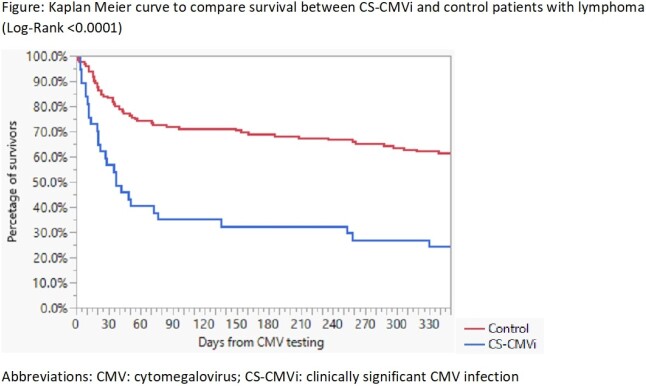

**Conclusion:**

CS-CMVi in lymphoma patients is associated with poor outcomes including oncologic outcomes. Further analysis to better define patients with lymphoma at high-risk for CS-CMVi should be studied for future preventative strategies.

**Disclosures:**

**Fareed Khawaja, MBBS**, MEDSCAPE: Honoraria|Viracor: Grant/Research Support **Roy F. Chemaly, MD/MPH**, Eurofins-VViracor: Grant/Research Support|Karius: Advisor/Consultant

